# Near-Space Wide-Area and High-Resolution Imaging System Design and Implementation

**DOI:** 10.3390/s23146454

**Published:** 2023-07-17

**Authors:** Zhanchao Wang, Min Huang, Lulu Qian, Yan Sun, Xiangning Lu, Wenhao Zhao, Zixuan Zhang, Guangming Wang, Yixin Zhao

**Affiliations:** 1 Aerospace Information Research Institute, Chinese Academy of Sciences, Beijing 100094, China; 2 Key Laboratory of Computational Optical Imaging Technology, Chinese Academy of Sciences, Beijing 100094, China; 3 School of Optoelectronics, University of Chinese Academy of Sciences, Beijing 100094, China

**Keywords:** near space, high resolution, optical design, imaging system

## Abstract

The near-space atmosphere is thin, and the atmospheric refraction and scattering on optical observation is very small, making it very suitable for wide-area and high-resolution surveillance using high-altitude balloon platforms. This paper adopts a 9344 × 7000 CMOS sensor to obtain high-resolution images, generating large-field-of-view imaging through the swing scanning of the photoelectric sphere and image stitching. In addition, a zoom lens is designed to achieve flexible applications for different scenarios, such as large-field-of-view and high-resolution imaging. The optical design results show that the camera system has good imaging quality within the focal length range of 320 mm–106.7 mm, and the relative distortion values at different focal lengths are less than 2%. The flight results indicate that the system can achieve seamless image stitching at a resolution of 0.2 m@20 km and the imaging field of view angle exceeds 33°. This system will perform other near-space flight experiments to verify its ultra-wide (field of view exceeding 100°) high-resolution imaging application.

## 1. Introduction

Aerial remote sensing is a comprehensive detection technology that uses optical, radar, and other technologies to obtain ground information through carrier platforms, such as manned aircraft, unmanned aerial vehicles, balloons, etc. It is flexible, low-cost, has a better vibration environment compared to airborne platforms, and is widely used in disaster monitoring, topographic mapping, military reconnaissance, and other fields [1,2,3].

Wide-area and high-resolution imaging technology is an important means of obtaining large, wide, and high-resolution ground information, and is also a key development direction in the field of remote sensing imaging in various countries around the world [4,5,6]. Near space generally refers to the space between 20 km and 100 km above the ground. The atmosphere in the adjacent space is thin, and the influence of water vapor, atmospheric refraction, and scattering on optical observation is very small, and it is very suitable for wide area surveillance [7].

As the most important indicators to measure the performance of imaging systems, width and resolution are two mutually constraining aspects of the technology. Wide-area and high-resolution imaging systems have broad application requirements, and researchers all over the world have proposed various new imaging structures and special methods to achieve wide-field-of-view and high-resolution imaging, including high-resolution camera scanning, multi-camera imaging, biomimetic optical imaging, and sensor array splicing imaging. High-resolution camera scanning imaging utilizes a high-resolution camera to scan and capture a large number of images, which can then be concatenated to obtain high-resolution images with a wide field of view. This method is a mature technology with high engineering reliability and the ability to freely adjust the imaging field of view and resolution, and it has other advantages due to its lightweight structure and simple operation control [8,9,10]. Multi-camera combined imaging indicates that the imager uses multiple imaging devices to capture spatial scenes at the same time, and each adjacent imaging device has a certain field of view overlap, a wide-field-of-view high-resolution image is obtained through the stitching of captured images; this method eliminates the rotating scanning mechanical structures and solves the time delay problem of the single camera scanning and shooting method [11,12]. The biomimetic optical imaging system is similar to multi-camera combination imaging, with the main difference being the design of the optical lens. It consists of dozens or even thousands of stacked or juxtaposed small imaging components with its wide-field-of-view decomposition, compact structure, and good multi-target fast parallel detection and tracking functions [13,14]; however, the pixel number of a single sensor still cannot meet practical application requirements. Therefore, it is necessary to integrate and splice multiple sensors to obtain larger pixel scale detectors. This solution has a high implementation cost and requires high splicing accuracy, and it is mainly used in large astronomical telescopes [15,16,17].

The aerial high-resolution camera is based on an optical system with a large aperture and long focal length. Under the condition of maintaining the spatial resolution, an increase in the imaging width will result in a rapid increase in the complexity, volume, and weight of the system, which are limited by the loading capacity of the platform and the attitude control capability. High-precision pose control and motion compensation technology have become key technologies that need to be addressed in the development process of large, wide, and high-resolution aerial camera systems.

In this paper, a high-resolution camera is designed and used for fine imaging of ground objects, and wide coverage is achieved through swing scanning by a photoelectric sphere. Due to the significant reduction in the field of view required for single imaging, it is easier to meet the constraints of camera volume and weight. The swing scanning achieves large area coverage by swinging the optical axis of the high-resolution camera along the wingspan direction. During the scanning process, the camera performs multiple exposure actions to obtain high-resolution images of different orientations in the wingspan direction. At the same time, the camera system follows the platform forward to achieve coverage in the flight direction, and then forms a large range of high-resolution images through stitching methods.

According to different image registration methods, image stitching is generally divided into three types: grayscale-based stitching methods, transform domain-based stitching methods, and feature-based stitching methods. The grayscale-based registration method is used to select a template in a fixed window from the reference image, search from the image to be registered, and use some evaluation method as the registration measurement to find the optimal registration position. The main stitching algorithms include the cross-correlation method, mutual information method, block matching method, ratio matching method, and grid matching method [18,19,20,21]. The mosaic method based on the transform domain is used to carry out some transformation on the image. The parameters of this mathematical transformation are separated by the method of parameter separation, and then the invariant is constructed. After matching the transformation model, the subsequent mosaic process is carried out. The main algorithms include wavelet transform, phase correlation, trace transform, and polar coordinate transform [22,23,24,25]. The feature-based stitching method is used to extract certain invariant features, such as interest points, lines, textures, etc., then match and calculate the transformation parameters between images, and finally complete the stitching. Feature-based stitching is currently the most widely used method, including Moravec, Harris, SIFT, SURF, ASIFT, GLOH, PCASIFT, and other algorithms [26,27,28,29,30].

The second part of this article mainly introduces the design of the wide-area and high-resolution imaging system. Firstly, the optical design of a high-resolution camera is introduced, mainly including the optical design, imaging quality analysis, thermal analysis, and tolerance analysis—the mechanical and electronic design of the camera are also introduced. Secondly, this paper introduces the design of the stable platform and the design of the swing scanning method. The third part of this article mainly introduces the flight verification results, including the image stitching results and high-resolution results.

## 2. Wide-Area and High-Resolution System Design

**Figure 1:**
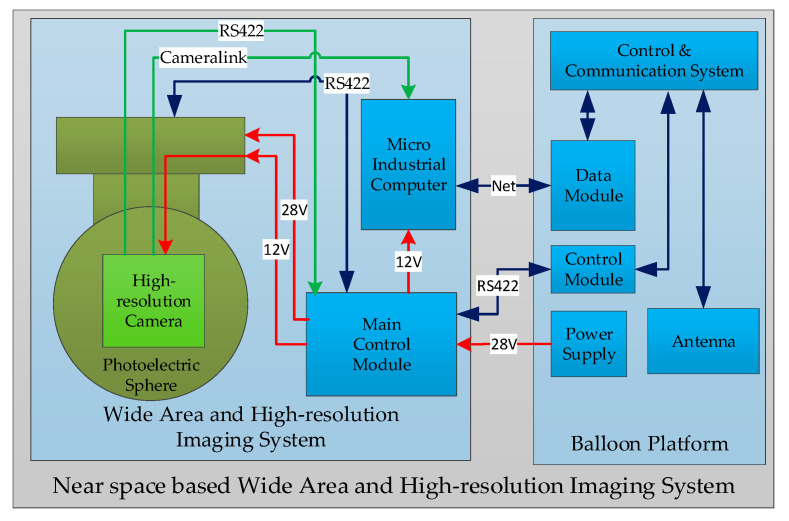
Near-space-based wide-area and high-resolution imaging system.

The near-space ball-borne wide-area and high-resolution imaging system mainly consists of a photoelectric sphere, a high-resolution camera, and a control system. The high-resolution camera is installed inside the photoelectric sphere and achieves high-resolution imaging with a large field of view through the rotation of the photoelectric sphere. The control system mainly generates the image collection and maintains control of the high-resolution camera, as well as the motion control of the photoelectric sphere. The composition diagram of the system is shown in Figure 1.

When the system is powered on, the high-resolution camera and the micro-industrial computer start working. The camera image data is sent to the micro-industrial computer automatically, and the micro-industrial computer stores the image data at the specified address. This address is set as a shared address, and the image data can be captured through the Internet. The main control module can set the parameters of the camera, such as the camera focal length, exposure time, and the pitch and azimuth angles of the photoelectric sphere. It is also possible to control the power up/down of the photoelectric sphere and the camera.

The ground console sends commands through the RS422 serial port, and the command data is transmitted to the public IP through the serial server. The ball-mounted serial port server obtains data from the public IP through satellite–Internet communication and sends commands to the main control module through the RS422 serial port. The main control module collects information, such as the angle and attitude of the photoelectric sphere, transmits it to the onboard serial port server through the RS422 serial port, and then transmits it to the public IP through satellite communication.

The main indicators of the system are shown in Table 1.

Section 2.1 mainly introduces the design and analysis of high-resolution cameras, including their optical design and analysis, thermal analysis, tolerance analysis, and structural and electronic design. Section 2.2 introduces the design of the photoelectric sphere and swing imaging schemes.

### 2.1. High-Resolution Camera Design

#### 2.1.1. Optical Analysis

**Figure 2:**
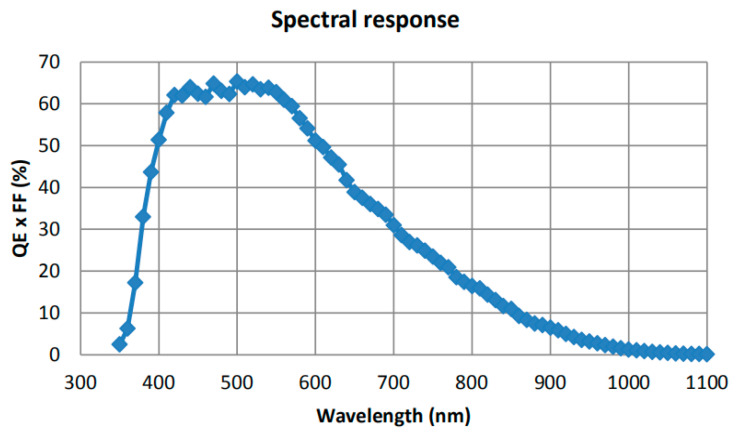
GMAX3265 Quantum Efficiency Curve.

This paper uses the GMAX3265 CMOS image sensor developed by Gpixel. The sensor has 65 million pixels, and the size of each pixel size is 3.2 μm. Its characteristics consist of a large pixel array, low readout noise, and a global shutter function. The main technical indicators are shown in Table 2 and the quantum efficiency curve is shown in Figure 2.

In order to improve the adaptability and performance of the high-resolution camera, the optical part is designed as a zoom lens. The optical system needs to design a set of high-resolution imaging lenses, and the primary optical parameters are calculated according to the requirements, as shown in Table 3.

#### 2.1.2. Optic System Design

**Figure 3:**
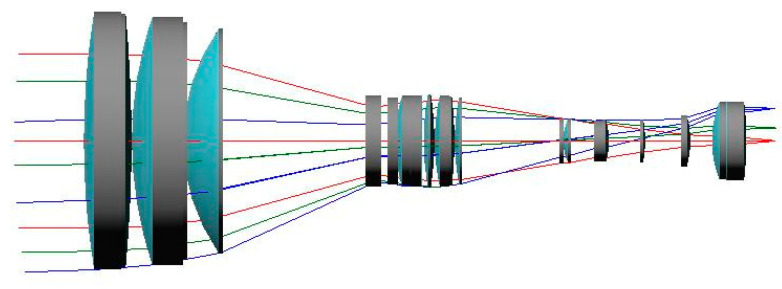
Optical path structure of the lens.

For the purpose of high-resolution imaging, the long focal length of the optical system is relatively large. Considering the limitations of the photoelectric sphere platform on load size in this project, the design process needs to compress the lens structure size as much as possible. Through the complex design of the initial structure, a three-fold zoom optical system with a long focal length of 320 mm, a short focal length of 106.7 mm, and F/# 4.3 was ultimately obtained. The optical path structure is shown in Figure 3, including two moving components. The movement of the zoom moving component from the short to long focal length is 33.1 mm, and the compensation moving component from the short to long focal length is 27.5 mm. The total length of the optical path is 310 mm, with a maximum aperture of 90 mm. There are 20 optical lenses, with a total weight of less than 1.8 kg.

The evaluation of the imaging quality of an optical system mainly considers the modulation transfer function (MTF), point plot, and distortion curve. According to the task requirements, the imaging quality of the optical system on the five preset focal segments are examined separately.

1. f = 320 mm

**Figure 4:**
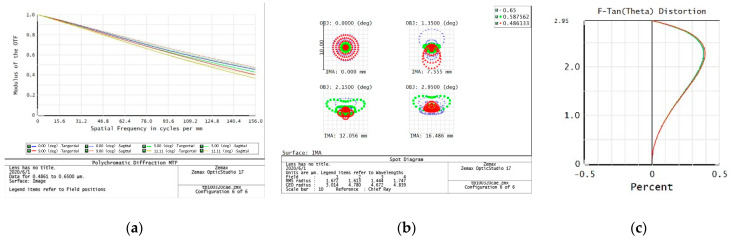
320 mm analysis result: (**a**) Full color MTF curve; (**b**) Full color dot plot curve; (**c**) Focal length distortion curve.

From the data of the telephoto endpoint of the optical system, the maximum RMS radius of the diffuse spot within the field of view is about 1.8 μm, as shown in Figure 4, which is less than a pixel size. From the MTF curve, the system has ideal image quality. At a Nessler frequency of 156 lp/mm, the full field MTF is close to 0.4, corresponding to an optical angular resolution of:

(1) $$\sigma =\frac{1}{N_{q}f}=20\;\mu \mathrm{rad}$$

When the resolution requirement of 20 μrad is met, the relative distortion curve of the system is as follows—with a maximum relative distortion of less than 0.4% in the full field of view.

2. f = 260 mm

**Figure 5:**
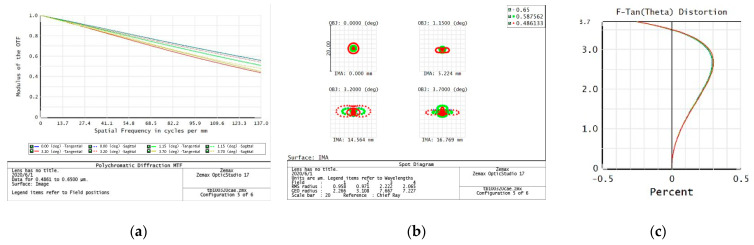
260 mm analysis result: (**a**) Full color MTF curve; (**b**) Full color dot plot curve; (**c**) Focal length distortion curve.

From the data of the telephoto endpoint of the optical system, the maximum RMS radius of the diffuse spot within the field of view is about 2 μm, as shown in Figure 5, which is less than a pixel size. From the MTF curve, the system has ideal image quality. At a Nessler frequency of 137 lp/mm, the full field MTF is close to 0.4, corresponding to an optical angular resolution of:

(2) $$\sigma =\frac{1}{N_{q}f}=28\;\mu \mathrm{rad}$$

When the resolution requirement of 28 μrad is met, the relative distortion curve of the system is as follows—with a maximum relative distortion of less than 0.3% in the full field of view.

3. f = 213 mm

**Figure 6:**
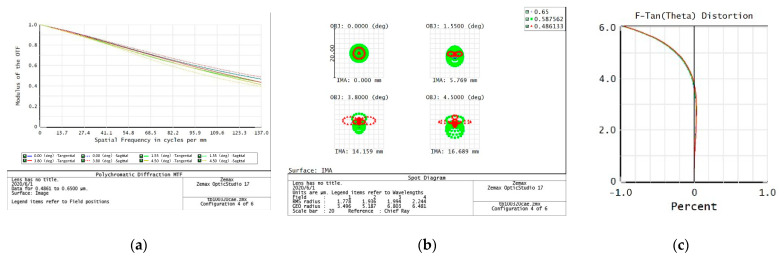
213 mm analysis result: (**a**) Full color MTF curve; (**b**) Full color dot plot curve; (**c**) Focal length distortion curve.

From the data of the telephoto endpoint of the optical system, the maximum RMS radius of the diffuse spot within the field of view is about 2.3 μm, as shown in Figure 6, which is less than a pixel size. From the MTF curve, the system has ideal image quality. At a Nessler frequency of 137 lp/mm, the full field MTF is close to 0.4, corresponding to an optical angular resolution of:

(3) $$\sigma =\frac{1}{N_{q}f}=34\;\mu \mathrm{rad}$$

When the resolution requirement of 35 μrad is met, the relative distortion curve of the system is as follows—with a maximum relative distortion of less than 0.5% in the full field of view.

4. f = 160 mm

**Figure 7:**
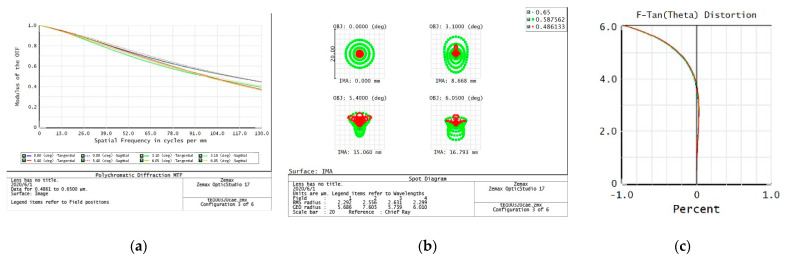
160 mm analysis result: (**a**) Full color MTF curve; (**b**) Full color dot plot curve; (**c**) Focal length distortion curve.

From the data of the telephoto endpoint of the optical system, the maximum RMS radius of the diffuse spot within the field of view is about 2.6 μm, as shown in Figure 7, which is less than a pixel size. From the MTF curve, the system has ideal image quality. At a Nessler frequency of 130 lp/mm, the full field MTF is close to 0.4, corresponding to an optical angular resolution of:

(4) $$\sigma =\frac{1}{N_{q}f}=48\;\mu \mathrm{rad}$$

When the resolution requirement of 50 μrad is met, the relative distortion curve of the system is as follows—with a maximum relative distortion of less than 1.0%.

5. f = 106.7 mm

**Figure 8:**
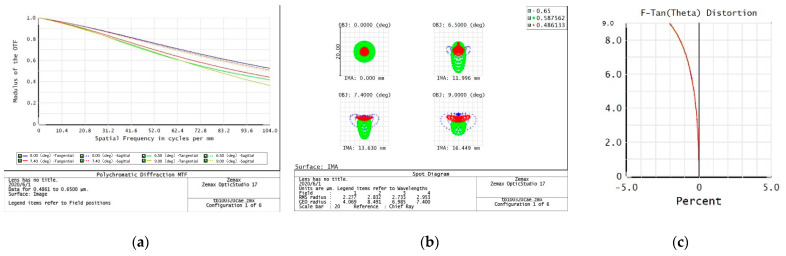
106.7 mm analysis result: (**a**) Full color MTF curve; (**b**) Full color dot plot curve; (**c**) Focal length distortion curve.

From the data of the telephoto endpoint of the optical system, the maximum RMS radius of the diffuse spot within the field of view is about 3.0 μm, as shown in Figure 8, which is less than a pixel size. From the MTF curve, the system has ideal image quality. At a Nessler frequency of 104 lp/mm, the full field MTF is close to 0.4, corresponding to an optical angular resolution of:

(5) $$\sigma =\frac{1}{N_{q}f}=90\;\mu \mathrm{rad}$$

When the resolution requirement of 90 μrad is met, the relative distortion curve of the system is as follows—with a maximum relative distortion of less than 2.0% in the full field of view.

#### 2.1.3. Thermal Analysis

**Figure 9:**
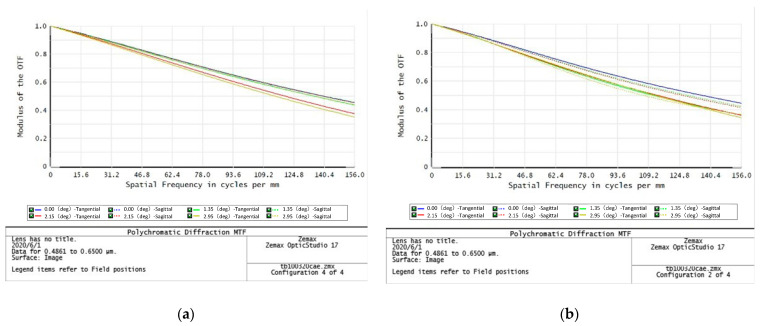
Transfer function diagram of 320 mm telephoto after focusing compensation: (**a**) +60 °C; (**b**) −45 °C.

When the temperature environment changes, the image quality of the optical system will change due to the influence of temperature defocusing. The reason for this result is that the temperature effect of ultra-low chromatic aberration glass is obvious, making the focal plane position of the system more sensitive to temperature. The continuous zoom lens uses a front motion group for temperature compensation to eliminate the influence of temperature effects. Within the temperature range of +60 °C to −45 °C, the transfer function curve remains basically constant, as shown in Figure 9.

#### 2.1.4. Tolerance Analysis

For the optical system that has been designed, a tolerance analysis is conducted to fully consider the impact of processing and assembly on the imaging quality of the system. The optical lens group adopts the centering machining process, and the tolerances are selected based on the experience values of medium- and high-precision objective lenses.

In order to predict the comprehensive effect of all tolerances during the overall assembly process of the system, a Monte Carlo method was used to analyze the influence of tolerances based on the design software. The MTF value of the optical system was used as the image quality evaluation index, and the spatial frequency was selected based on the optical angular resolution of the short and long focal ends.

**Figure 10:**
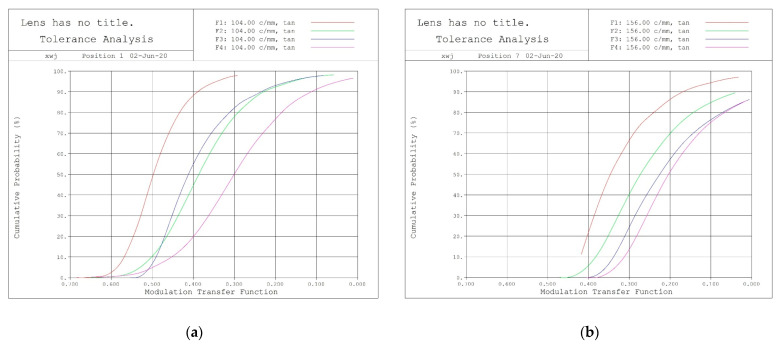
Tolerance analysis results: (**a**) 106.7 mm; (**b**) 320 mm.

As is shown in the tolerance analysis result in Figure 10, according to the selected component processing and assembly tolerances, the lens can be processed and adjusted, with a 60% probability of achieving a full field of view range with a selected spatial frequency MTF greater than 0.15, and a high one-time pass rate, which can meet production requirements.

#### 2.1.5. Electrical and Mechanical Design

**Figure 11:**
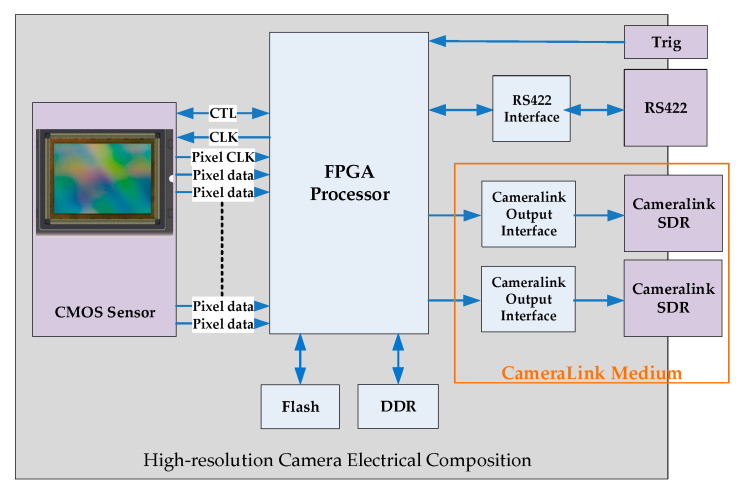
Electrical composition of the camera.

The electronic system of a high-resolution camera consists of CMOS sensors, an image data processing interface, signal processor, power converters, control input, and image output interfaces. The composition of the camera system is shown in Figure 11.

**Figure 12:**
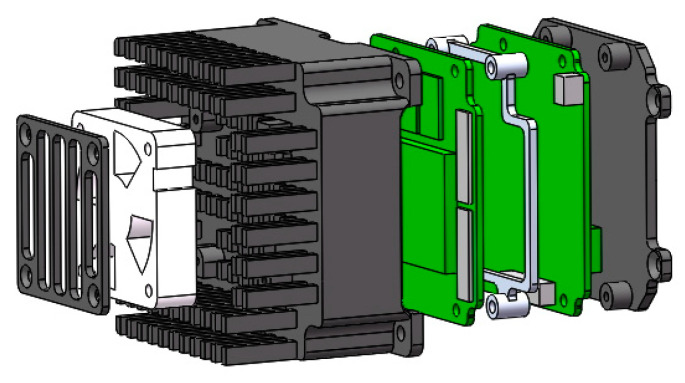
Electrical structure of the camera.

The development of the detector has gone through multiple stages, including scheme design, structural design, PCB design, prototype debugging, and overall testing. The PCB circuit stack installation structure of the detector is shown in Figure 12.

**Figure 13:**
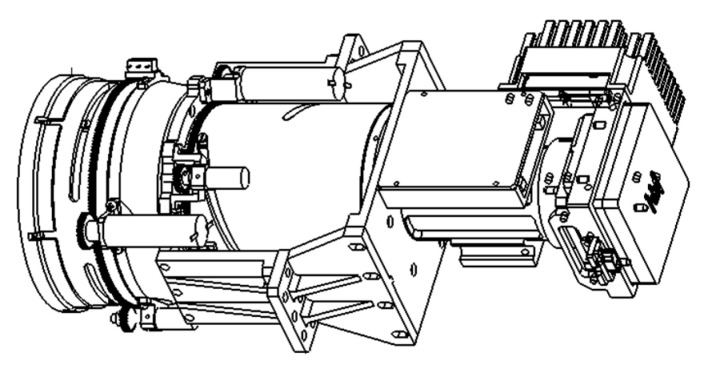
Mechanical structure of the camera.

To ensure the implementation of optical design, the mechanical structure of the camera mainly includes three parts: a front mirror component, a focusing mirror component, and a detector component, as shown in Figure 13.

The front mirror component contains the five optical lenses of the camera. In order to meet the requirements for the co-axiality of the optical path, structural stability, and ease of assembly and adjustment, the assembly adopts an integral cylindrical structure. The front mirror component mainly includes the mirror tube, protective cover, pins, compression rings and washer fixing parts, spacers, mirror boxes, trimming pads, etc. The lens barrel mainly provides support and positioning for various optical lenses and is fixedly connected to the focusing lens component.

The focusing mirror component includes the four optical lenses of this camera. Considering the optical design requirements and structural simplification, the component is designed as an I-type straight-through lens tube structure; this component is in the middle of the entire camera, and square flange plates are placed at both ends to connect the front mirror component and detector component. Since there is a lens in the optical system that is sensitive to changes in ambient temperature, a focusing module is placed in the focusing mirror assembly. The focusing module consists of a stepping motor, big and small gears, and photoelectric travel switches—and its corresponding structural parts. Through the design of the number of teeth, modulus, and pressure angle of the big and small gears, a reasonable reduction ratio is optimized. With the help of the electronic control system, the optical focusing requirements are met.

The temperature environment during camera operation fluctuates greatly, and a higher temperature will affect the imaging quality of the CMOS sensor. To ensure that the CMOS sensor operates in an ideal temperature environment, thermal conductivity cooling is used to timely dissipate the heat generated by the CMOS sensor during operation. There is a thermal conductivity plate on its back, with one end fixed to the back of the CMOS chip and an insulation pad added at the fixed position. The other end is led out, and then heat is transmitted to the thermal control component to achieve conducting cooling. The thermal conductivity plate is made of red copper (T3) with high thermal conductivity. To improve the thermal conductivity efficiency and increase the contact area, a combination of mechanical pressing and thermal adhesive bonding is used to fix it. The CMOS base is made of titanium alloy material, which has a certain strength and low thermal conductivity, which is conducive to the thermal control of CMOS chips.

### 2.2. Photoelectric Sphere and Swing Imaging Design

**Figure 14:**
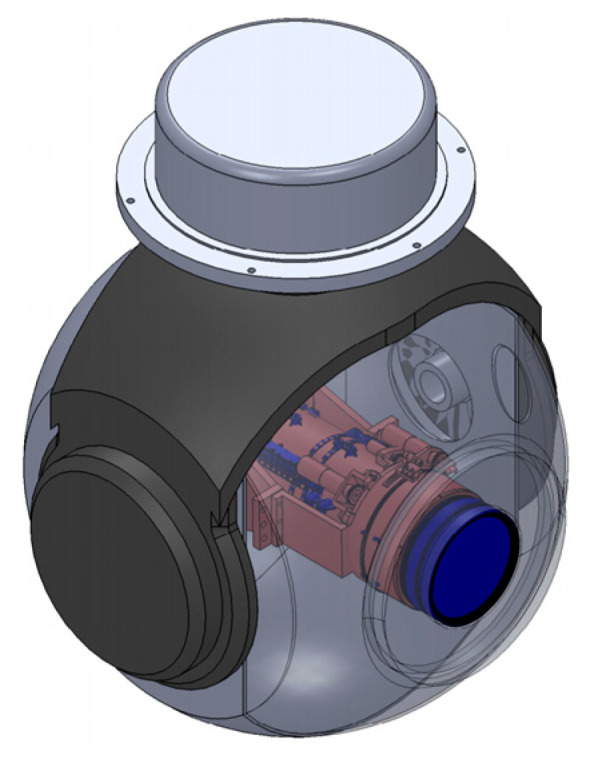
The installation of the high-resolution camera in the photoelectric sphere.

The design of the photoelectric sphere adopts a two axis and two frame stable system structure, and the high-resolution camera is loaded into the pitch frame. The design of the photoelectric platform adopts a spherical structure. On the one hand, the aerodynamic resistance of the spherical structure is smaller than that of other structures (plate, square, diamond, etc.) under the same conditions (same speed, same windward area); on the other hand, the product has good symmetry. During flight, when the azimuth and pitch frames move, the changes in their angular positions cause small changes in the driving torque, making system control easy to implement; thirdly, under certain conditions of stable platform rotation space, the internal space contained by the sphere is larger than that of other structures, and its load-bearing capacity is strong. The installation of the high-resolution camera in the photoelectric sphere is shown in Figure 14.

**Figure 15:**
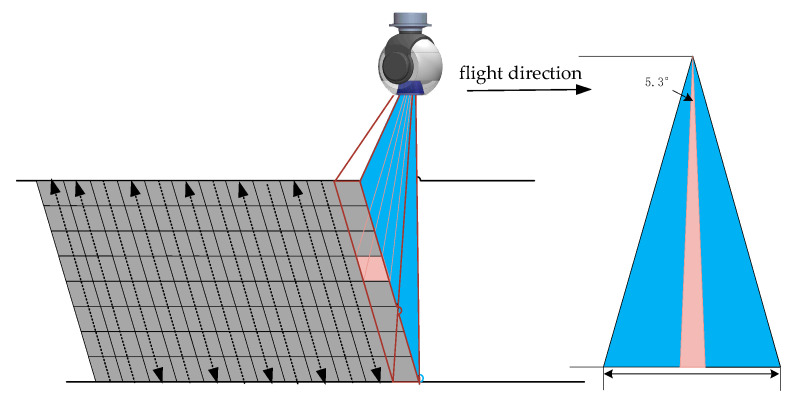
Mode of photoelectric sphere and high-resolution camera.

In the process of wide-area high-resolution imaging, the high-resolution camera operates in telephoto mode, with a photoelectric sphere azimuth angle of 90°—perpendicular to the flight direction—and an initial pitch angle of −90°, indicating ground imaging. The pitch direction of the photoelectric sphere provides different imaging fields of view for high-resolution cameras to take photos through steeper motions, as shown in Figure 15. Due to the very slow flight speed of high-altitude balloons, which is about 10 m/s, there is no need to consider the image shift in the direction of flight when performing swing imaging.

Due to the significant distortion caused by large angle squint, in order to reduce the level of image distortion, the sweep width coverage is 33.3°, the step angle is 3.5°, and the overlap rate in the span direction is 35%. The optical axis roll angle of each image frame in a single scanning cycle is uniformly distributed within the width range, and the values are shown in Table 4.

The SIFT algorithm is very robust in complex geometric images and radiative conditions and is currently the most commonly used registration algorithm. The process of SIFT is mainly divided into four stages: feature point detection, key point localization, direction allocation, and feature point description. Firstly, the scale space of the image is constructed by establishing a Difference of Gaussians (DOG) pyramid to extract feature points and obtain the first layer image through Gaussian blur.

(6) $$L\left(x,y,\sigma \right)=G\left(x,y,\sigma \right)\times I\left(x,y\right)$$

$I\left(x,y\right)$ is the original image, and $G\left(x,y,\sigma \right)$ is the Gaussian kernel, while $\left(x,y\right)$ are the pixel coordinates, and σ is the scale factor, wherein the larger the σ, the clearer the image contour, and the smaller the σ, the more obvious the image details. The Gaussian kernel function is:

(7) $$G\left(x,y,\sigma \right)=\frac{1}{2\pi \sigma }e^{-\frac{x^{2}+y^{2}}{2\sigma^{2}}}$$

The difference of Gaussians pyramid performs the DOG operation between two adjacent layers of images, which is shown as:

(8) $$D\left(x,y,\sigma \right)=\left[G\left(x,y,k\sigma \right)-G\left(x,y,\sigma \right)\right]\times I\left(x,y\right)=L\left(x,y,k\sigma \right)-L\left(x,y,\sigma \right)$$

When locating key points, it is necessary to filter out low-contrast extreme points and edge responses. It is necessary to obtain a 2 × 2 Hessian matrix by taking the derivative of the Gaussian convolution formula.

(9) $$H=\left[\begin{array}{cc} D_{xx} & D_{xy}\\ D_{yx} & D_{yy} \end{array}\right]$$

The direction matching of feature points mainly estimates the direction of neighboring pixels of key points through the grayscale histogram of the image.

(10) $$m\left(x,y\right)=\sqrt{{\left(L\left(x+1,y\right)-L\left(x-1,y\right)\right)}^{2}+{\left(L\left(x,y+1\right)-L\left(x,y-1\right)\right)}^{2}}$$

(11) $$\theta \left(x,y\right)=tan^{-1}\left(L\left(x,y+1\right)-L\left(x,y-1\right)\right)/\left(L\left(x+1,y\right)-L\left(x-1,y\right)\right)$$

$m\left(x,y\right)\;$ where represents the gradient value and $\theta \left(x,y\right)$ represents the gradient direction.

## 3. Results

**Figure 16:**
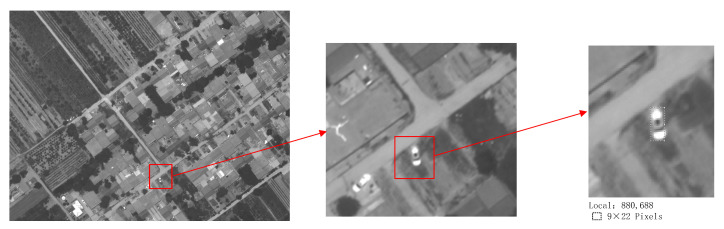
Result of the flight experiment image resolution.

In May 2022, a flight experiment was conducted to verify the functionality of the wide-area high-resolution imaging system. The focal length of the camera is set to 320 mm to achieve a resolution of 0.2 m@20 km, as shown in Figure 16. The car in Figure 16 occupies approximately 9 × 22 pixels, based on the resolution, and the actual size is approximately 1.8 m × 4.4 m, which is consistent with the actual situation.

**Figure 17:**
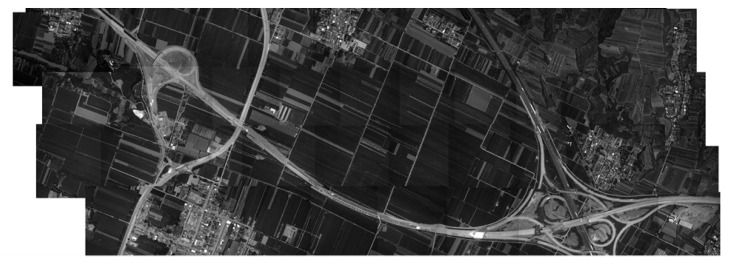
Result of the flight experiment image stitching.

We adopted the SIFT method to achieve the image stitching, where the coverage is 33.3° and the image stitching result is shown in Figure 17, indicating that the wide-area and high-resolution imaging system has a good image stitching effect, and the overlap rate meets the seamless stitching requirements.

## 4. Conclusions

This paper adopts a 64 M pixels CMOS sensor to obtain high-resolution images and generates a large-field-of-view image through the swing scanning of the photoelectric sphere. The flight results indicate that the system can achieve seamless image stitching at a resolution of 0.2 m@20 km and coverage of 33.3°.

In addition, the high-resolution camera adopts zoom lenses (focal length range of 320 mm–106.7 mm) to achieve flexible applications. For application scenarios with ultra-wide (field of view exceeding 100°) high-resolution imaging, ground resolution sharply decreases with an increase in the scanning angle, and the resolution of the edge area is about half of the center area, seriously affecting the image stitching effect. The zoom swing scanning wide-area and high-resolution imaging system in this article can adjust the camera focal length in real-time during the swing scanning process, ensuring that the imaging resolution remains unchanged. It provides a new solution for ultra-wide and high-resolution imaging. This system will be used in other near-space flight experiments to verify its application.

## Figures and Tables

**Table 1:** Main performance indicators.

Item	Data
Resolution	0.2 m@20 km
Azimuth angle of photoelectric sphere	−180° to 180°
Pitch angle of photoelectric sphere	−140° to 140°

**Table 2:** GMAX3265 Image Sensor Index Parameter Table.

Item	Data
effective number of pixels	9344 × 7000
pixel size	3.2 μm × 3.2 μm
photosensitive area	29.9 mm × 22.4 mm
image mode	monochrome camera
image data bit	12 bit (max)
maximum of frame rate	31 frame/s
dynamic range	66 dB
peak quantum efficiency	65.3%@500 nm
number of saturated electrons	>9.0 ke−
read noise	2.0 e−
dark current	5.3 e−/p/s@40 °C
nyquist frequency	156 lp/mm
consumption	<2.1 W

**Table 3:** High-resolution camera optical parameters.

Item	Data
long focal length	$f_{L}^{\prime }=20000\;m\frac{3.2\;\mu m}{0.2\;m}=320\;\mathrm{mm}$
short focal length	$f_{S}^{\prime }=\frac{320\;\mathrm{mm}}{3}=106.7\;\mathrm{mm}$
horizontal field of long focal	$2\omega =2\times \arctan\left(\frac{29.9\;\mathrm{mm}}{2\;\times \;f_{L}^{\prime }}\right)\approx 5.3\;{}^{\circ}$
vertical field of long focal	$2\omega =2\times \arctan\left(\frac{22.4\;\mathrm{mm}}{2\;\times \;f_{L}^{\prime }}\right)\approx 4{}^{\circ}$
horizontal field of short focal	$2\omega =2\times \arctan\left(\frac{29.9\;\mathrm{mm}}{2\;\times \;f_{S}^{\prime }}\right)\approx 16.0{}^{\circ}$
vertical field of short focal	$2\omega =2\times \arctan\left(\frac{22.4\;\mathrm{mm}}{2\;\times \;f_{S}^{\prime }}\right)\approx 12{}^{\circ}$

**Table 4:** Photoelectric sphere rotation angle.

Position Number	Rotation Angle (°)	Horizontal Angle Coverage (°)
1	−14.0	−16.65~−11.35
2	−10.5	−13.15~−7.85
3	−7.0	−9.65~−4.35
4	−3.5	−6.15~−0.85
5	0	−2.65~2.65
6	3.5	0.85~6.15
7	7.0	4.35~9.65
8	10.5	7.85~13.15
9	14.0	11.35~16.65

## Data Availability

Not applicable.
